# Identification of possible candidate genes regulating Sjögren's syndrome-associated autoimmunity: a potential role for *TNFSF4 *in autoimmune exocrinopathy

**DOI:** 10.1186/ar2560

**Published:** 2008-11-25

**Authors:** Cuong Q Nguyen, Janet G Cornelius, Lauren Cooper, Jonathan Neff, Joann Tao, Byung Ha Lee, Ammon B Peck

**Affiliations:** 1 Department of Oral Biology, University of Florida, 1600 SW Archer Road, Gainesville, FL 32610, USA; 2 Department of Pathology, Immunology & Laboratory Medicine, University of Florida, 1600 SW Archer Road, Gainesville, FL 32610, USA; 3 Center for Orphan Autoimmune Disorders, College of Dentistry, University of Florida, 1600 SW Archer Road, Gainesville, FL 32610, USA

## Abstract

**Introduction:**

Sjögren syndrome (SjS) is a systemic autoimmune disease in which an immunological attack primarily against the salivary and lacrimal glands results in the loss of acinar cell tissue and function, leading to stomatitis sicca and keratoconjunctivitis sicca. In recent years, two genetic regions, one on chromosome 1 (designated autoimmune exocrinopathy 2 or *Aec2*) and the second on chromosome 3 (designated autoimmune exocrinopathy 1 or *Aec1*) derived from nonobese diabetic (NOD) mice, have been shown to be necessary and sufficient to replicate SjS-like disease in nonsusceptible C57BL/6 mice.

**Methods:**

Starting with the SjS-susceptible C57BL/6-derived mouse, referred to as C57BL/6.NOD-*Aec1Aec2*, we generated a large set of recombinant inbred (RI) lines containing portions of *Aec2 *as a means of identifying more precisely the genetic elements of chromosome 1 responsible for disease development.

**Results:**

Disease profiling of these RI lines has revealed that the SjS susceptibility genes of *Aec2 *lie within a region located at approximately 79 ± 5 cM distal to the centromere, as defined by microsatellite markers. This chromosomal region contains several sets of genes known to correlate with various immunopathological features of SjS as well as disease susceptibility genes for both type 1 diabetes and systemic lupus erythematosus in mice. One gene in particular, tumor necrosis factor (ligand) superfamily member 4 (or *Ox40 ligand*), encoding a product whose biological functions correlate with both physiological homeostasis and immune regulations, could be a potential candidate SjS susceptibility gene.

**Conclusions:**

These new RI lines represent the first step not only in fine mapping SjS susceptibility loci but also in identifying potential candidate SjS susceptibility genes. Identification of possible candidate genes permits construction of models describing underlying molecular pathogenic mechanisms in this model of SjS and establishes a basis for construction of specific gene knockout mice.

## Introduction

Sjögren syndrome (SjS) is a chronic, systemic, human autoimmune disease in which an immunological attack initially against the salivary and lacrimal glands results, respectively, in dry mouth (stomatitis sicca) and dry eye (keratoconjunctivitis sicca) disease(s) [1-3]. Despite efforts to define the genetic, environmental, and immunological bases of SjS, the underlying etiology of this disease remains ill defined. In attempts to better define the nature of SjS autoimmunity, a variety of mouse models exhibiting various aspects of SjS have been studied extensively [4]. One of the more intensively studied models of SjS is the nonobese diabetic (NOD) mouse [5-9]. Based on disease profiling of various congenic partners and gene knockout lines of NOD, we have proposed that the development and onset of SjS-like disease in these mice can be divided into at least three distinct consecutive phases [10-19]. In phase 1, a number of aberrant physiological and biochemical activities, thought to result from a genetically based retarded salivary gland organogenesis and increased acinar cell apoptosis, occur prior to and independent of detectable autoimmunity. In phase 2, believed to result from the glandular cell injury of phase 1, small numbers of macrophages and dendritic cells are attracted to the exocrine gland where these sentinel cells recruit T and B lymphocytes that form lymphocytic foci (LF), some of which histologically appear as germinal centers. In phase 3, the onset of clinical disease as defined by salivary and lacrimal gland secretory dysfunction occurs, possibly resulting first from the production of autoantibodies that interfere with the neural-acinar cell signaling pathways and then from progressive loss of acinar cell mass hastened by the action of effector T cells.

A genetic predisposition for development and onset of SjS-like disease in NOD mice has also been defined. First, SjS-like disease in these mice appears independent of or only weakly associated with major histocompatibility complex (MHC) class I and class II genes [10,20], thus mimicking SjS in humans. This can be seen by the fact that the congenic strain, NOD.B10-*H2*^*b*^, in which the NOD MHC *I-A*^*g*7^*Idd1 *diabetes susceptibility locus was replaced by the MHC *I-A*^*b *^locus [20], continued to show SjS-like disease, including salivary and lacrimal gland dysfunction. Second, replacing *Idd *loci other than *Idd1 *(for example, *Idd9*, *Idd10*, and *Idd13*) resulted in the identification of *Idd3 *on chromosome 3 and *Idd5 *on chromosome 1 as critical genetic regions for development of SjS-like disease in NOD mice [10]. In a reverse approach, introducing both *Idd3 *and *Idd5 *derived from NOD mice into SjS-nonsusceptible C57BL/6 mice resulted in a severe SjS-like disease, confirming the contributions of these two genetic loci to the development and onset of SjS [21]. Furthermore, the preclinical nonimmune aspects manifested in phase 1 of the disease appeared to associate with the *Idd5 *locus (referred to as autoimmune exocrinopathy 2 or *Aec2*), whereas the immunological aspects of the disease manifested in phases 2 and 3 of the disease appeared to associate with *Idd3 *(referred to as *Aec1*). This recently generated mouse strain is referred to as C57BL/6.NOD-*Aec1Aec2*. While the pathophysiological and immunological aspects may not be linked solely to one or the other genetic region (as originally proposed [22]), the complete disease profile requires genes within both of these genetic loci.

For years, identification of candidate genes associated with autoimmune diseases such as T1D [23] or systemic lupus erythematosus [24] in animal models has been providing invaluable data on delineating the genetic components of these diseases, now translating to the human disease. These studies have formed a template for our current efforts to identify the SjS susceptibility loci and candidate genes underlying SjS which, in this respect, have lagged behind many other autoimmune diseases. Although our initial work defined the *Aec1 *and *Aec2 *genetic regions present in C57BL/6.NOD-*Aec1Aec2 *mice as being an approximately 48.5-cM centromeric region on chromosome 3 and an approximately 73.3-cM telomeric region on chromosome 1, respectively [10], the size of these regions precluded identification of candidate genes. Subsequently, we shortened *Aec1 *to an approximately 19.2-cM region in the first studied recombinant inbred (RI) line, C57BL/6.NOD-*Aec1R01Aec2 *[9]. For the present study, we generated a set of new RI lines that further demarcate the boundaries of *Aec2*. These new C57BL/6.NOD-*Aec1Aec2R(n) *RI lines identify not only a much shorter *Aec2 *sublocus at position 79 cM of chromosome 1, but also potential candidate SjS susceptibility genes on which to build hypothetical models that can be tested for validating possible pathogenic molecular mechanisms of SjS-like disease.

## Materials and methods

### Animals

C57BL/6.NOD-*Aec1Aec2R(n) *and C57BL/6.NOD-*Aec1R(n)Aec2R(n) *mice were generated by crossing C57BL/6.NOD-*Aec1Aec2 *mice with C57BL/6J mice purchased from The Jackson Laboratory (Bar Harbor, ME, USA). The F1 heterozygotes were screened for the presence of crossover events within the *Aec1 *and/or *Aec2 *genetic regions by microsatellite marker genotyping. Individual mice indicating a crossover in *Aec2 *were bred with a C57BL/6J mouse to produce *Aec2 *crossover heterozygous male and female offspring that were then used to produce F2 generations. Mice of the F2 generations were screened for a male and female homozygous for the crossover chromosome. Once an appropriate homozygous recombinant founder pair was identified, the RI line was maintained via a single line of descent.

All RI lines were bred and maintained under specific pathogen-free conditions in the animal facility of Animal Care Services of the University of Florida (Gainesville, FL, USA). Both male and female mice 4 to 24 weeks of age were used in the following studies. All mice received water and food *ad libitum*. Blood samples were collected while the mice were anesthetized with isoflurane. Euthanasia was carried out by cervical dislocation after anesthetization with isoflurane or 100% CO_2_. Studies described herein were approved by the University of Florida Institutional Animal Care and Use Committee.

### Genotyping

To determine the genetic status of each offspring, DNA was prepared using the DNeasy Tissue Kit (Qiagen Inc., Valencia, CA, USA) from a small tail snip taken between 2 and 4 weeks of age just prior to weaning. Each DNA sample was used as a template in polymerase chain reaction amplification with D1mit primers covering the *Aec2 *genetic region. Microsatellite markers that differentiated genes derived from NOD mice from those derived from C57BL/6J mice were chosen. Primer sequences for the microsatellite markers were based on sequences available from The Jackson Laboratory and purchased from Integrated DNA Technologies (IDT, Coralville, IA, USA).

### Measurement of saliva flow rates

To measure stimulated flow rates of saliva, individual mice were weighed and given an intraperitoneal injection of 100 μL of a mixture containing isoproterenol (0.02 mg/1 mL of phosphate-buffered saline [PBS]) and pilocarpine (0.05 mg/1 mL of PBS). Saliva was collected for 10 minutes from the oral cavity of individual mice using a micropipette starting 1 minute after injection of the secretagogue. The volume of each saliva sample was measured. The saliva samples were then frozen at -80°C until analyzed.

### Histology

Male and female C57BL/6.NOD-*Aec1Aec2R(n) *mice were euthanized at various ages as indicated in the text. Submandibular and lacrimal glands were surgically removed from each mouse and placed in 10% phosphate-buffered formalin for 24 hours. Fixed tissues were embedded in paraffin and sectioned at 5-μm thickness. Paraffin-embedded sections were de-paraffinized by immersing in xylene, followed by dehydrating in ethanol. The tissue sections were prepared and stained with hematoxylin and eosin dye (Histology Tech Services, Inc., Gainesville, FL, USA). Stained sections were observed at × 100 magnifications for glandular structure and leukocyte infiltration. To detect and determine leukocytic infiltrations in salivary and lacrimal glands, a single histological section per gland per mouse was examined by two individuals blinded to the RI lines. LF, defined as aggregates of greater than 50 leukocytes, were quantified for each section.

### Detection of anti-nuclear autoantibodies in the sera

Anti-nuclear autoantibodies (ANAs) in the sera of mice were detected using an ANA screening kit (Immuno Concepts, Sacramento, CA, USA). Sera were tested at dilutions of 1:40, 1:80, and 1:160. Presented in this paper, however, are data from testing sera at 1:40 dilutions. In brief, HEp-2 fixed substrate slides were overlaid with the appropriate mouse serum. Slides were incubated for 30 minutes at room temperature in a humidified chamber. After three washes for 5 minutes with PBS, the substrate slides were covered with Alexa 594-conjugated goat anti-mouse IgG (H/L) (Invitrogen Corporation, Carlsbad, CA, USA) diluted 1:50 for 30 minutes at room temperature. After three washes, nuclear fluorescence was detected by fluorescence microscopy at × 100 magnification.

### Modeling of biological pathways using Pathway Studio

To model biological pathways from selected genes located within the redefined *Aec2 *genetic region, Pathway Studio version 5.0 software (Ariadne Genomics, Rockville, MD, USA) and the ResNet mammalian database were used. Functions of selected genes within the two genetic regions and known SjS-related genes were first verified from the ResNet mammalian database and then imported into Pathway Studio to visually construct molecular and biological interactions or relationships among the inputted genes.

### Statistical analyses

For this study, we have standardized both saliva and tear collections based on the body weight of the individual mice in an attempt to better control comparisons. We have incorporated this for mice of the C57BL/6 genetic background because, first, disease tends to occur in the C57BL/6 genetic background strains at an earlier age, often necessitating collections of saliva and tears when the mice are as young as 4 to 6 weeks of age and are less than half the size of adult mice, and, second, there are greater size differences between male and female mice during the time course studied. Statistical evaluations between saliva collections were determined by using the unpaired *t *test generated by GraphPad InStat software (GraphPad Software, Inc., San Diego, CA, USA). A two-tailed *P *value of less than 0.05 was considered significant.

## Results

### Genetic profiling of the recombinant inbred lines

**Figure 1:**
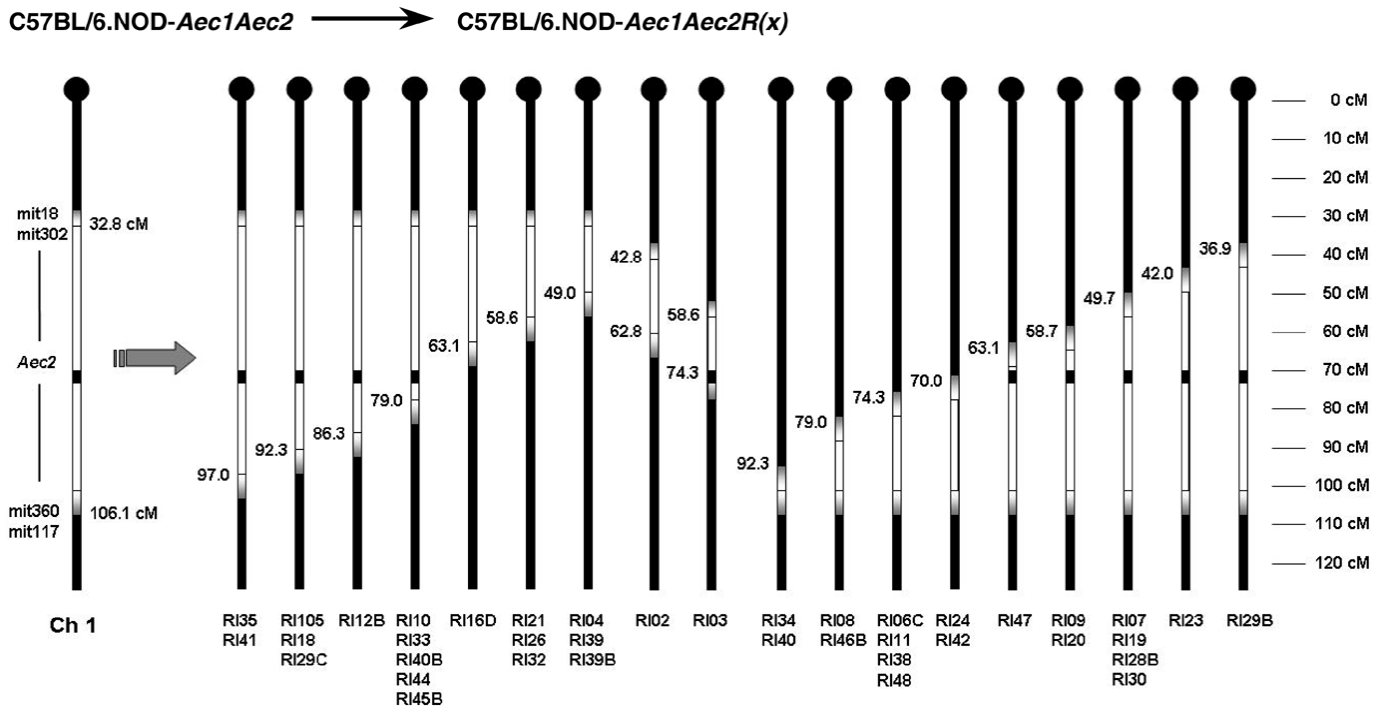
Map of chromosome 1 crossover points in C57BL/6.NOD-*Aec1Aec2R(n) *recombinant inbred (RI) mice. Thirty-nine RI lines are aligned to show the points of their individual crossovers in the *Aec2 *region of chromosome 1, as determined by D1mit microsatellite markers. Crossover frequencies are higher at approximately 49.7, 74.3, and 79.0 cM but are not considered hotspots for chromosomal 1 crossovers (NS: Not significant, * = p < 0.05, ** = p < 0.01, and *** = p < 0.001).

From an initial mating of C57BL/6J males with C57BL/6.NOD-*Aec1Aec2 *females, we identified 49 unique crossovers in *Aec2 *of chromosome 1, consisting of 33 lines with a single crossover in *Aec2 *and 16 lines with a crossover in both *Aec1 *and *Aec2*. In addition, 2 lines were established using mice with pre-existing double-crossovers in the *Aec2 *region (RI lines 02 and 03). During the subsequent inbreeding, we were successful in generating 39 new homozygous *Aec2 *RI lines. To map each genetic segment of the *Aec2 *region remaining within each of the 39 newly generated RI lines, we selected microsatellite markers spaced approximately 4 to 5 cM apart along chromosome 1. As presented in Figure 1, these new RI lines, taken together, define progressively smaller genetic segments of *Aec2 *derived from NOD mice and permit much finer mapping for SjS susceptibility loci. Although there are at least two regions on chromosome 1 (around positions 50 and 75 cM) that exhibited higher numbers of recombinant events, there do not appear to be any crossover hotspots.

### Disease profiling of the recombinant inbred lines

SjS-like disease in our NOD-derived mouse lines, including C57BL/6.NOD-*Aec1Aec2*, is characterized generally by three criteria [4], reflecting the objective criteria used to identify SjS in humans [25]. These are (a) the loss of saliva and tear flow rates over time, (b) the presence of LF in the salivary and lacrimal glands, and (c) the presence of ANAs in sera. To determine which of the RI C57BL/6.NOD-*Aec1Aec2R(n) *mice develop salivary gland dysfunction, temporal changes in saliva flow rates were determined for both male and female mice at an early age (7 ± 1 weeks) and then at a later age (22 ± 2 weeks). The number of mice examined for each new RI line was dependent on the number of offspring produced in the first few pregnancies following inbreeding.

**Figure 2:**
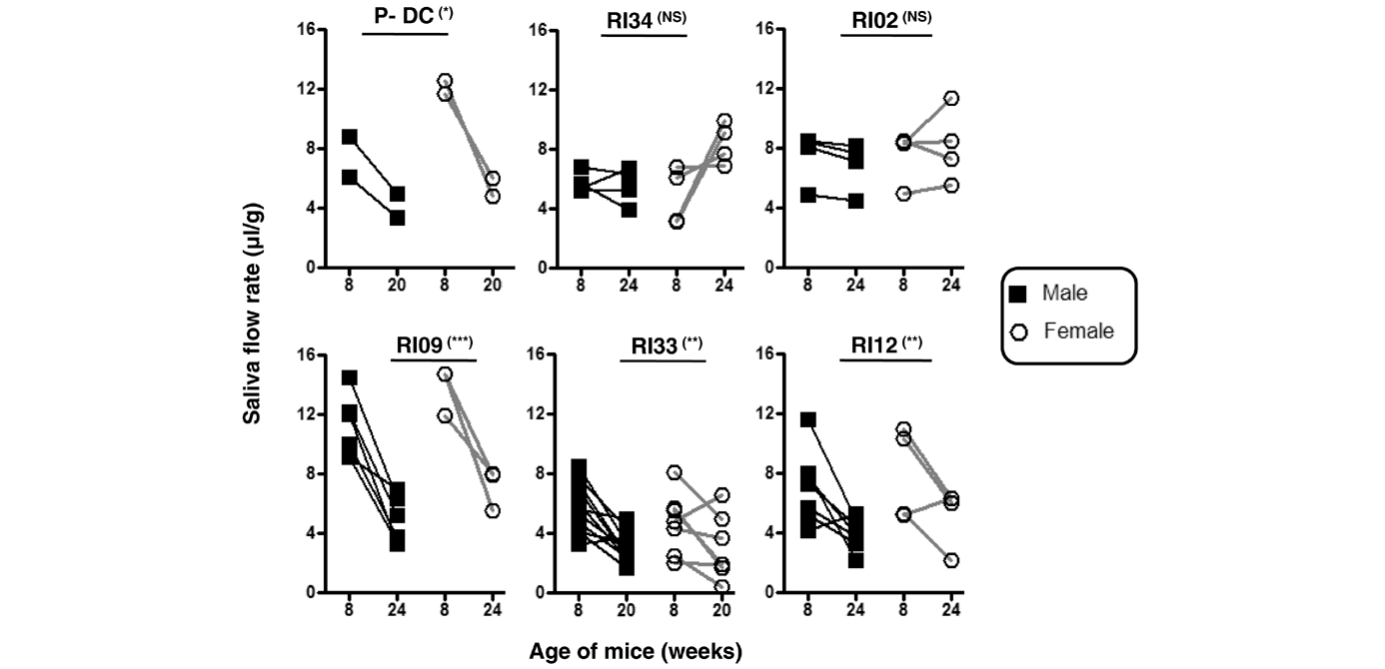
Differences in temporal loss of secretory function in various C57BL/6.NOD-*Aec1Aec2R(n) *mice. Male and female sibling mice of parental C57BL/6.NOD-*Aec1Aec2 *(P-DC) and C57BL/6.NOD-*Aec1Aec2R(n) *mice were injected with isoproterenol/pilocarpine, first at 8 weeks of age and then at 20 or 24 weeks of age, to stimulate saliva secretion. Saliva was collected from each mouse for 10 minutes starting 1 minute after injection of the secretagogue. The volume of each sample was measured and standardized relative to the weight of the mouse. Temporal reductions in saliva secretions, a marker for onset of clinical disease, were used to identify genetic regions containing genes necessary for development of salivary gland dysfunction and Sjögren syndrome. NS, not significant; RI, recombinant inbred.

Results indicate that the loss of secretory flow rates was clearly evident for several of the RI lines, thereby retaining the phenotype of parental C57BL/6.NOD-*Aec1Aec2 *mice, while a number of the RI lines also failed to show a loss of secretory activities, thereby indicating loss of the SjS-like disease phenotype. Selected yet representative data showing differences in salivary flow rates among the *Aec2 *RI lines are shown in Figure 2. For example, both male and female mice of RI lines RI09, RI33, and RI12, all of which retained the parental *Aec1 *region but carry various portions of *Aec2*, exhibited salivary gland dysfunction as measured by loss of salivary flow rates ranging generally between 35% and 60% as the mice aged from 8 to 20–24 weeks. These data are consistent with the decreases of saliva fluid volumes historically observed with NOD, NOD.B10-*H2*^*b*^, and C57BL/6.NOD-*Aec1Aec2 *mice [8,10,16,20,21]. In contrast, male and female RI mice of lines exhibiting little or no salivary gland dysfunction (for example, RI34 and RI02) generally showed slightly increased salivary flow rates over these same time frames, mimicking SjS-nonsusceptible parental C57BL/6J mice.

**Figure 3:**
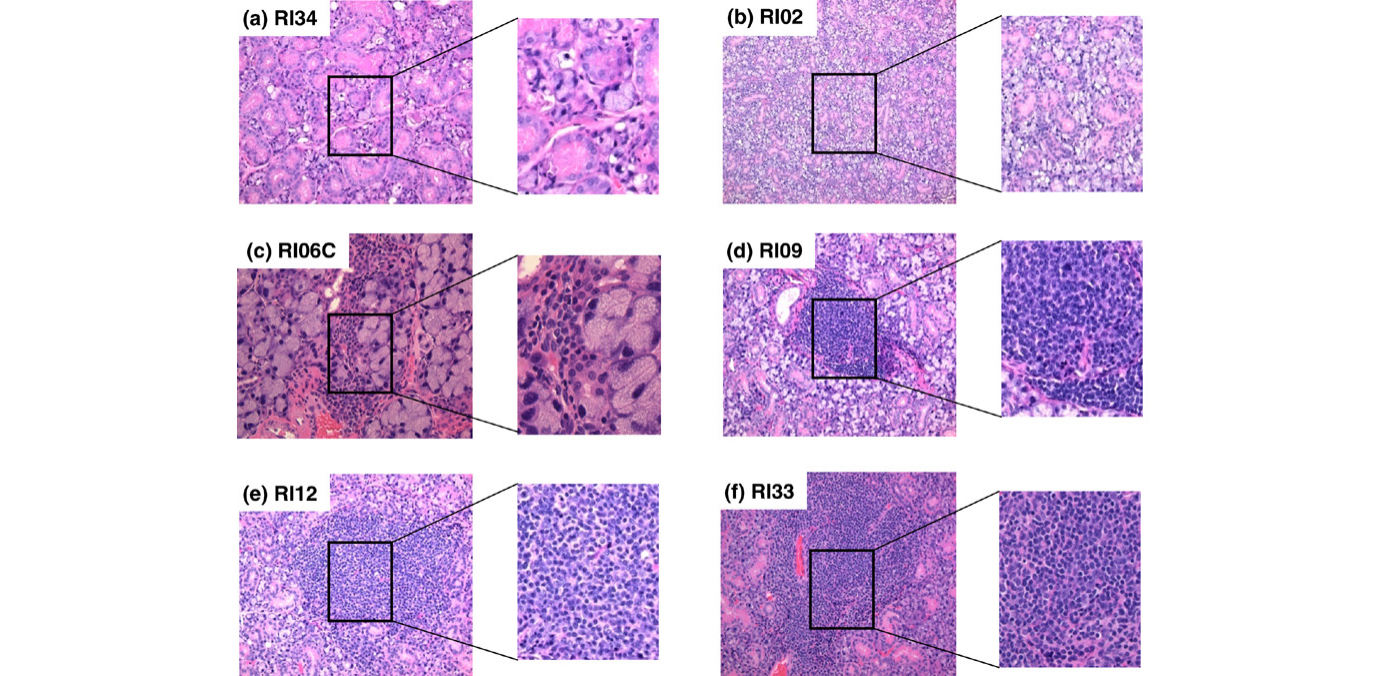
Histological characterization of sialadenitis of male and female C57BL/6.NOD-*Aec1Aec2R(n) *mice. Submandibular glands were freshly explanted from male and female C57BL/6.NOD-*Aec1Aec2R(n) *mice euthanized at 20 or 24 weeks of age. The glands were fixed in 10% formalin, embedded in paraffin, and sectioned and stained with hematoxylin and eosin (H&E) dye. Representative H&E-stained histological sections of submandibular glands of selected recombinant inbred (RI) lines are presented: **(a) **RI34, **(b) **RI02, **(c) **RI06C, **(d) **RI09, **(e) **RI12, and **(f) **RI33. Original images were taken at × 100 magnification, with inserts expanded to show structural detail.

**Figure 4:**
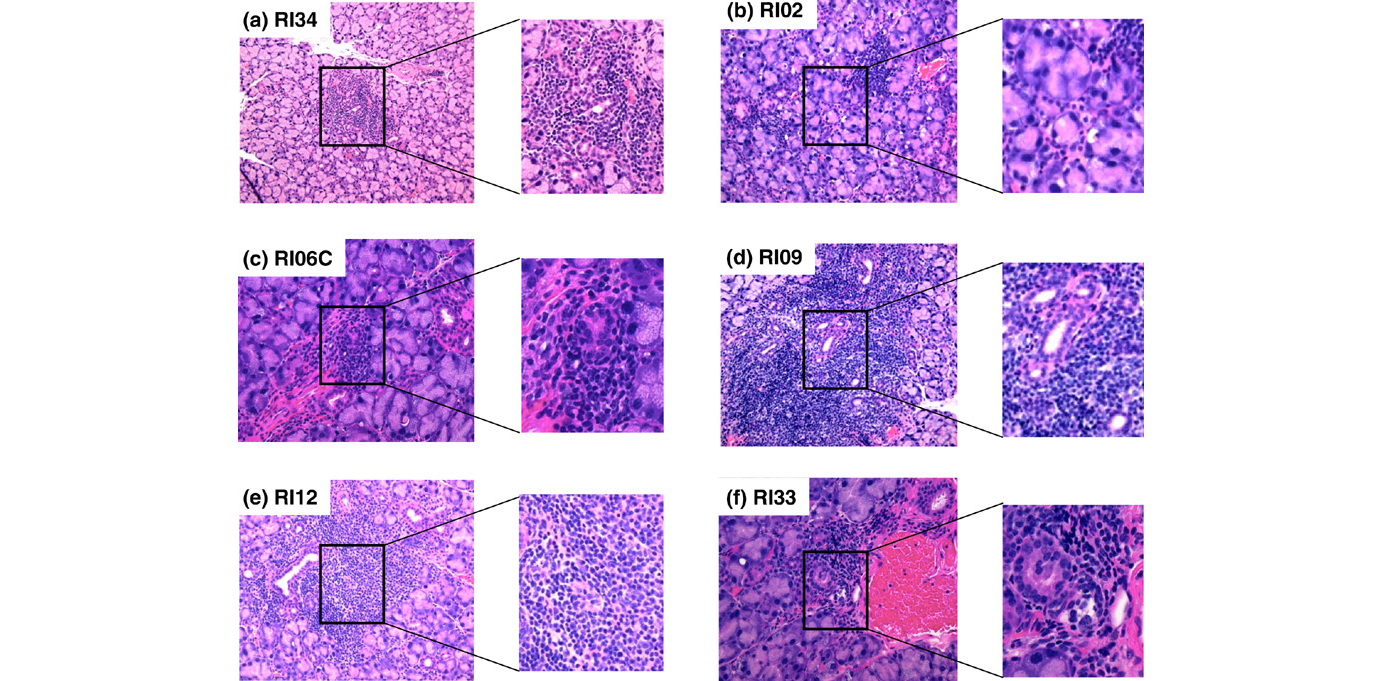
Histological characterization of dacryoadenitis of male and female C57BL/6.NOD-*Aec1Aec2R(n) *mice. Submandibular and lacrimal glands were freshly explanted from male and female C57BL/6.NOD-*Aec1Aec2R(n) *mice euthanized at 20 or 24 weeks of age. The glands were fixed in 10% formalin, embedded in paraffin, and sectioned and stained with hematoxylin and eosin (H&E) dye. Representative H&E-stained histological sections of lacrimal glands of selected recombinant inbred (RI) lines are presented: **(a) **RI34, **(b) **RI02, **(c) **RI06C, **(d) **RI09, **(e) **RI12, and **(f) **RI33. Original images were taken at × 100 magnification, with inserts expanded to show structural detail.

Although the number of LF present in minor salivary gland biopsies of SjS patients often does not correlate directly with disease or severity of disease, both SjS patients and NOD-derived mice exhibiting SjS-like disease typically present with LF. As presented in Figures 3 and 4, histological examinations revealed the presence of LF in the submandibular and extraorbital lacrimal glands, starting at 8 to 12 weeks of age in all of the anticipated disease-susceptible RI strains (for example, RI06, RI09, RI33, and RI12). In contrast, no LF or at most only a relatively few, smaller LF were seen in the glands of RI34 and RI02 mice, correlating with their normal salivary flow rates. Interestingly, in addition to the lymphocytic infiltrates, increased levels of lipid deposits could be seen in the submandibular and lacrimal glands of several RI lines with onset of disease (data not shown). Quantification of LF in the salivary and lacrimal glands showing the relative differences in SjS-susceptible (RI06, RI09, RI12, and RI33) versus SjS-nonsusceptible (RI02 and RI34) RI lines is provided in Table 1.

**Table 1:** Quantification of lymphocytic foci in the salivary and lacrimal glands of mice from several representative C57BL/6.NOD-*Aec1Aec2R(n) *recombinant inbred lines

RI line	Age, weeks	Submandibular glands					Lacrimal glands				
			
		Number of mice		Positive		Average number of LF	Number of mice		Positive		Average number of LF
							
		Male	Female	Number	Male and female		Male	Female	Number	Male and female	
RI 34	20 and 24	6	7	3	23%	1.0 ± 0.0	6	7	7	54%	1.7 ± 0.4
RI 02	20 and 24	4	7	0	0%	0.0 ± 0.0	6	8	4	29%	1.5 ± 0.3
RI 06	20	4	ND	3	74%	4.0 ± 1.7	4	ND	4	100%	5.8 ± 4.1
RI 09	21 and 22	7	7	11	79%	3.0 ± 0.6	7	7	6	43%	2.8 ± 0.7
RI 12	24	7	4	5	45%	2.4 ± 0.7	7	4	10	91%	2.8 ± 0.6
RI 33	20 and 21	9	7	10	63%	2.2 ± 1.0	9	7	13	81%	6.5 ± 4.5

LF, lymphocytic foci; ND, not done; RI, recombinant inbred.

**Figure 5:**
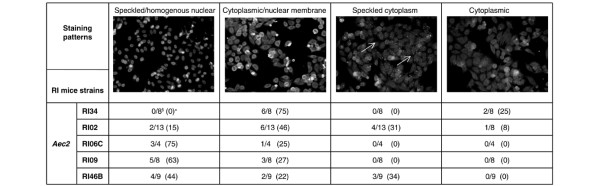
Detection of anti-nuclear autoantibodies in sera of C57BL/6.NOD-*Aec1RAec2R(n) *mice. Serum samples obtained from C57BL/6.NOD-*Aec1Aec2R(n) *mice were diluted 1:40 and incubated with HEp-2 fixed substrate slides for 30 minutes at 25°C in a humidified chamber. The slides were then developed with Alexa 594-conjugated goat anti-mouse IgG and viewed by fluorescence microscopy at × 100 magnification. Examples of speckled/homogenous staining of the nucleus (left panel), cytoplasmic/nuclear membrane staining (left center panel), speckled/cytoplasmic staining (right center panel), and cytoplasmic staining (right panel) were observed. The numbers of individual sera tested and the percentages of positive sera from a sampling of recombinant inbred (RI) lines exhibiting each of the patterns are listed. ^§^Number of mice showing positive staining pattern over total. *Percentage of mice showing positive staining pattern. Aec, autoimmune exocrinopathy.

The presence of ANAs, in particular anti-SS-A/Ro and anti-SS-B/La in the sera of human patients, is one parameter in the diagnosis of clinical SjS. Concomitantly with the appearance of mononuclear leukocytes within the salivary and lacrimal glands of parental C57BL/6.NOD-*Aec1Aec2 *mice, increasing numbers and levels of detectable serum autoantibodies are also detected [26-29]. To identify ANAs in the sera of RI C57BL/6.NOD-*Aec1Aec2R(n) *mice, both male and female mice were serially bled between 6 and 24 weeks of age (until euthanasia) and the sera were collected and tested on HEp-2 cells. As presented in Figure 5, a number of different patterns of ANA staining, including speckled/homogenous nuclear, cytoplasmic/nuclear membrane, speckled cytoplasm, and cytoplasmic staining, were detected in the sera from different RI lines. Cytoplasmic with nuclear membrane and cytoplasmic staining patterns appeared to be more prevalent in sera from SjS-nonsusceptible RI lines (RI02 and RI34), whereas sera from SjS-susceptible RI lines such as RI06C, RI09, and RI46B produced predominantly speckled/homogenous staining patterns. In general, a majority of sera from mice classified as SjS-susceptible RI lines produced ANA staining patterns observed with sera from parental C57BL/6.NOD-*Aec1Aec2 *mice and not NOD mice [9]. This difference between the C57BL/6 background-derived mice versus the NOD and NOD.B10.*H2*^*b *^mice suggests that the ANA staining pattern is not disease-specific and that the genetic background plays an important role in which ANAs are synthesized. Furthermore, the speckled pattern of staining in these RI lines appears to be characteristic of the staining observed with anti-SS-A/Ro and anti-SS-B/La antibodies [30]. At the same time, the cytoplasmic punctate staining is characteristic of the staining observed with antibodies against GW bodies [31]. Confirmation of whether these antibodies are reactive with SS-A/Ro, SS-B/La, and/or GW bodies is currently ongoing.

### Redefining the Sjögren syndrome susceptibility *Aec2 *genetic region

**Figure 6:**
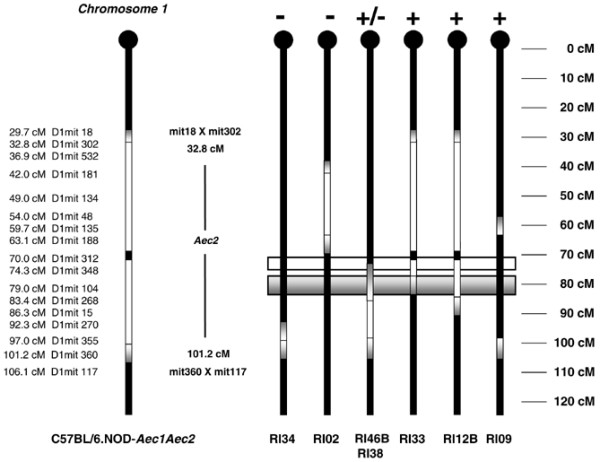
Redefining the boundaries for the *Aec2 *Sjögren Syndrome (SjS) susceptibility genetic locus. Based on the disease profiling of the C57BL/6.NOD-*Aec1Aec2R(n) *recombinant inbred (RI) lines, the boundaries of the *Aec2 *genetic region containing SjS susceptibility genes have been temporarily reset to position 79 ± 5 cM of chromosome 1 (shaded gray gradient rectangular box). Possible quantitative trait loci genes may reside a few centimorgans centromeric to this region (unshaded rectangular box).

Based on the disease profiling data, we are now able to tentatively identify a small segment (subregion) of *Aec2 *containing genes essential and sufficient for development and onset of SjS-like disease associated with NOD and NOD-derived mice. As shown in Figure 6, the primary (or candidate) SjS susceptibility gene(s) on chromosome 1 lay within a genetic region around 79 ± 5 cM. SjS susceptibility genes within this sublocus must be coexpressed with the NOD-derived genes of the *Aec1 *region of chromosome 3 in order to induce a clinical disease. Not surprisingly, however, this redefined *Aec2 *subregion contains multiple genes already shown to correlate with human and mouse SjS as well as several additional autoimmune diseases in mice. These genes provide a basis for developing hypothetical models of molecular mechanisms underlying SjS, as discussed below.

## Discussion

In the present study, in which the specific goal was to redefine (and narrow) the boundaries of the *Aec2 *genetic region on chromosome 1 known to predispose NOD and NOD-derived lines of mice to SjS, we generated a large set of new RI lines (n = 39) and examined each line for its SjS-like disease profile. Disease profiles obtained with the C57BL/6.NOD-*Aec1Aec2R(n) *RI lines indicate that the *Aec2 *genetic region of C57BL/6.NOD-*Aec1Aec2 *mice, postulated to regulate primarily the pathophysiological and biochemical abnormalities that subsequently result in the activation of the autoimmune attack against the submandibular and lacrimal glands [10], is a single subregion mapping to the telomeric portion of chromosome 1 located at approximately 79 ± 5 cM. However, penetrance and severity of SjS-like disease may be further influenced by genes located within a few centimorgans on the centromeric side of this region, possibly pointing to SjS-associated quantitative trait loci (QTL) genes. Although the size of the redefined *Aec2 *region remains relatively large for identification of individual candidate SjS susceptibility genes, the genes residing within this subregion can be grouped into four functionally clustered sets, each suspected previously of involvement in SjS susceptibility. These are (a) endogenous viruses and oncogenic genes, (b) Fas/FasL-associated apoptosis, (c) T_H_17-associated activities, and (d) fatty acid, lipid, lipoprotein, and cholesterol homeostasis. However, perhaps the most obvious aspect is the fact that this redefined *Aec2 *region contains the QTL-*Ath1 *region containing some 10 genes, including tumor necrosis factor ligand superfamily member 4 (*Tnfsf4 *or *Ox40L*) and *Tnfsf6 *(*Fasl*).

Within the first set, several viral/oncogenic genes, such as *Emv38 *(endogenous ecotropic MuLV-38), *Kras-2-rs1 *(Kirsten rat sarcoma oncogene-2, related sequence-1), *Xpr1 *(xenotropic/polytropic retrovirus receptor-1), and *Abl2 *(Abelson murine leukemia viral oncogene-2), are found in this redefined *Aec2 *subregion. In our earlier studies with NOD mice [12], we observed that high levels of interferon-gamma (INF-γ) were present in the salivary glands of neonate mice, suggesting an important role for INF-γ in the delayed development/proliferation of acinar tissue observed in the salivary glands of neonate NOD mice. While it is logical to conclude that induction of INF-γ may be a result of short-term viral infection during the preterm and early postpartum periods, what might cause a viral outbreak at this time point remains unknown. It could be hypothesized that this occurs due to the changes in maternal hormone levels at this time. Perhaps more interesting, however, this region contains the gene *Tnfsf6 *encoding the proapoptotic protein FasL. FasL has numerous functions but is mainly involved in regulating immune responses, apoptosis, and retinal cell programmed death [4]. During the early phase 1 period of SjS-like disease in NOD mice, both FasL and Fas are upregulated at both the gene and protein levels, and this increased expression of Fas/FasL corresponds to the observed increase in acinar cell apoptosis within the glands [32]. However, it remains speculative whether there might be an association between endogenous/exogenous viral infection and Fas/FasL activity in the salivary and lacrimal glands.

The redefined *Aec2 *subregion also contains several genes involved in autoimmunity and/or tumorgenesis, the latter being one clinical manifestation of SjS that occurs in a small subset of patients. Of interest, but not thought to be directly involved in the development and onset of SjS, is the presence of genes specific to the ocular/lacrimal gland etiology (for example, *Pdc *[phosducin], which is a protein of the retinal photoreceptors cells [33], and *Myoc *[myocilin], whose product interacts with olfactemedin involved in glaucoma [34]). However, whether any of these genes are related to SjS susceptibility and lacrimal gland disease or merely influence the secondary disease phenotypes often associated with SjS remains unknown. In contrast, one genetic element in this region that has a direct association with the immunopathology of SjS is the QTL gene *Cypr2 *(cytokine production 2) [35]. CYPR-2 is known to regulate levels of interleukin-10 (IL-10), an important cytokine that enhances the activity of B lymphocytes, and at the same time to regulate the functions of T_H_1 and T_H_17 cells [36]. Our recent microarray studies indicate that *Il10 *is not upregulated during the development of SjS in the C57BL/6.NOD-*Aec1Aec2 *mouse model [32], possibly indicating a lack of immune regulation by regulatory T (T_reg_) cells. If so, this lack of regulation by IL-10 would be consistent with the results of gene therapy studies in which injections of vectors expressing recombinant IL-10 reduced or suppressed clinical manifestations of SjS-like disease in both salivary and lacrimal glands of mice [37,38].

**Figure 7:**
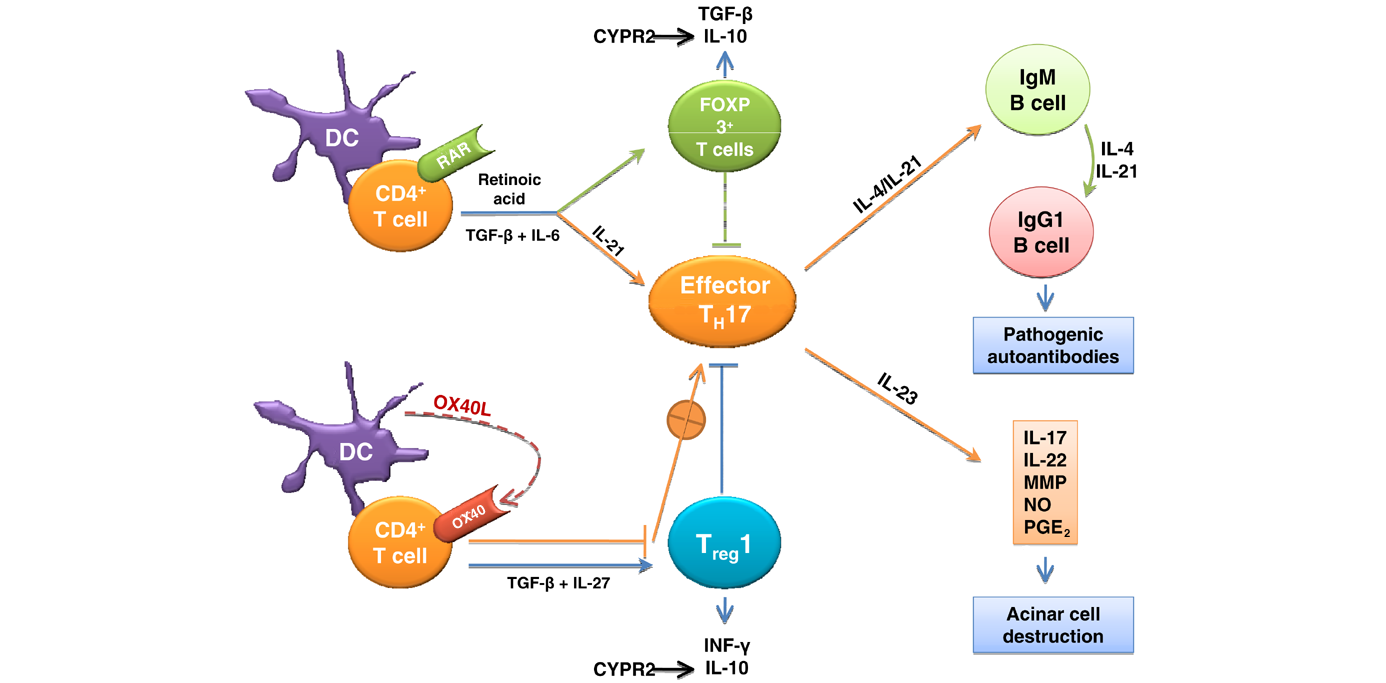
Proposed model for how OX40L:OX40 promotes autoimmunity in Sjögren syndrome-like disease of C57BL/6.NOD-*Aec1Aec2 *mice. Cellular interactions involved in the development of an autoimmune response against the salivary and lacrimal glands leading to loss of acinar tissue are presented. DC, dendritic cell; IL, interleukin; INF-γ, interferon-gamma; MMP, matrix metalloproteinase; NO, nitric oxide, PGE_2_, prostaglandin E_2_; TGF-β, transforming growth factor-beta; T_reg_, regulatory T (cell).

Maintaining sufficient regulation of immune responses to prevent development of an overt autoimmunity is no doubt dependent on a physiological balance between T_H_1, T_H_2, T_H_17, and T_reg _cell interactions. This cellular interaction appears to be highly influenced by OX40L encoded by the *Tnfsf4 *gene and this gene is located within the redefined *Aec2 *region. OX40L is expressed by a number of distinct cell populations, including activated dendritic cells [39]. OX40L is capable of functioning as an inhibitor of the maturation of T_reg_1 cells [40], a regulatory cell population normally producing IL-10 and INF-γ, which (in conjunction with IL-27) can inhibit the effector CD4^+ ^T_H_17 cells [41]. Reduced T_reg_1 cell function, therefore, results in a positive feedback for the generation/activation of effector CD4^+ ^T_H_17 cells (Figure 7). These effector T_H_17 cells produce predominantly IL-17, IL-21, and IL-22 plus factors like nitric oxide, matrix metalloproteinase, and prostaglandin E_2_, each of which is shown to play an important role in the immunopathophysiology of several autoimmune diseases, including SjS [41]. Thus, we hypothesize that the presence of T_H_17 cells in the salivary and lacrimal glands of C57BL/6.NOD-*Aec1Aec2 *mice, as well as SjS patients [42], indicates an imbalance in the T_H_17/T_reg_1 ratio favoring the T_H_17 population(s). A recent study indicates that retinoic acid can facilitate an increase in the numbers of Foxp3^+ ^T_reg _cells and simultenously inhibit the formation of effector T_H_17 cells [43]. Interestingly, we have found that expression of the retinoic receptors, *Rxr *(retinoid × receptor) and *Rar *(retinoic acid receptor), is downregulated in the lacrimal glands of C57BL/6.NOD-*Aec1Aec2 *mice [44]. This observation is again consistent with a potential problem in cellular homeostasis, especially at the level of macrophages, dendritic cells, and even production of the FOXP3^+ ^T_reg _cell populations whose differentiation and functional maturation are highly dependent on retinoic and fatty acid stimulation. As presented in Figure 7, there is a reciprocal maturation of effector CD4^+ ^T_H_17 and FOXP3^+ ^T_reg _cells dependent on the relative balance of IL-6 and transforming growth factor-beta influenced by retinoic acid. Thus, the intricacy and balance between OX40L, the retinoids, proinflammatory cytokines, and development of T_reg _cells appear to impact the potential development and onset of autoimmunity, which in SjS appears to favor activation of effector T_H_17 cells.

**Figure 8:**
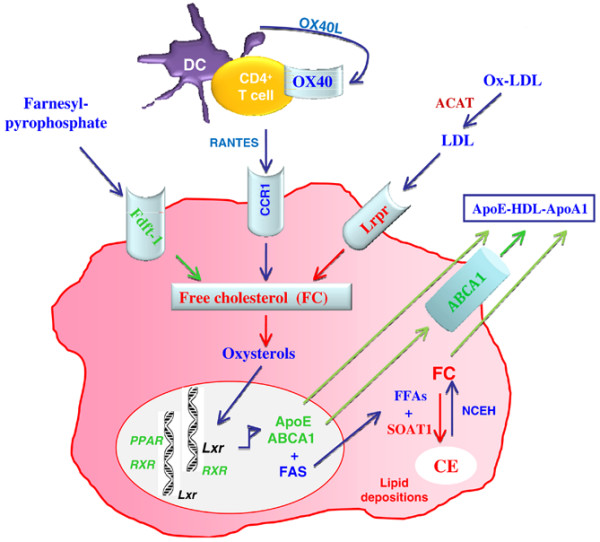
Proposed genetic predisposition for dysregulated homeostasis/transport of lipid, lipoprotein, cholesterol, and fatty acid metabolism leading to lipid depositions in the salivary and lacrimal glands of C57BL/6.NOD-*Aec1Aec2 *mice and Sjögren syndrome patients. Accumulation of free cholesterols (FCs) inside the cells resulted from increased uptake of low- and high-density lipid receptors. In addition, impairment of ABCA1 membrane transporter leads to the accumulation of cholesteryl esters (CEs) metabolized by sterol O-acyltransferase-1 (SOAT-1) using FCs and free fatty acids (FFAs). ABCA1, ATP-binding cassette, subfamily A [ABC1] member 1; ACAT, acyl-coenzyme A: cholesterol acyltransferase; ApoE, apolipoprotein E; DC, dendritic cell; Fdft-1, farnesyl diphosphate farnesyl transferase-1; HDL, high-density lipid; LDL, low-density lipid; Lrpr, low-density lipid-related protein receptor; NCEH, neutral cholesterol esters hydrolase; Ox-LDL, oxidized low-density lipid; PPAR, peroxisome proliferator activated receptor; RANTES, regulated on activation normal T cell expressed and secreted; RXR, retinoid × receptor. Adapted from [48].

Although several factors encoded by genes in the redefined *Aec2 *region may be involved in secondary manifestations of SjS, OX40L is the one factor that clearly stands out as a primary candidate gene underlying not only the recognized immune dysregulation, as presented above, but also the pathophysiological aberrations associated with chromosome 1 of the C57BL/6.NOD-*Aec1Aec2 *SjS model. In this redefined region, a common functional cluster of lipid, lipoprotein, cholesterol, and fatty acid regulatory and processing elements is found, including *Hdlq14 *(high-density lipoprotein QLT-14), *Hdlq5 *or *Apoa2 *(high-density lipoprotein QTL-5), *Gpa33 *(glycoprotein A33), *Cq1 *(cholesterol QTL-1), *Prdx6 *(peroxiredoxin), and (of special note) *Soat1 *(sterol O-acyltransferase-1). Involvement of lipids and fatty acids in the pathology of SjS has become a major focus of SjS research as lipid depositions [45] and changes in lipid rafts [46] appear to influence the pathology in both salivary and lacrimal glands. Furthermore, our recent genomic microarray studies – [44,47] (C.Q. Nguyen, S. Ashok, R.A McIndoe, J.X. She, B.H. Lee, A.B. Peck, unpublished data) – indicate that multiple genes involved in fatty acid, lipid, lipoprotein, and cholesterol homeostasis/transport are differentially expressed, corresponding with lipid deposits, dysfunctional dendritic cells, and onset of autoimmunity. As illustrated in Figure 8, various relationships between genes controlling free fatty acid, lipid, and lipoprotein homeostasis are directly or indirectly dependent on the activities of OX40L. As a consequence, imbalances in this homeostasis regulated in part by OX40/OX40L can result in widespread pathology, including inflammation and cell death. Based on differential gene expression data, there are major reductions in the levels of transcripts encoding FDFT-1 (farnesyl diphosphate farnesyl transferase-1), ABCA1 (ATP-binding cassette, subfamily A [ABC1] member 1), and the retinoic acid receptors, RRX and RAR. At the same time, increased levels of transcripts encoding the low- and high-density lipoprotein receptors, as well as SOAT-1, are observed. We hypothesize, therefore, that an imbalance occurs in the production of cholesterol and the increased level of cholesterol results in greater amounts being converted by SOAT-1 to cholesteryl esters that accumulate within the cells due to the downregulation or dysfunction of the lipid transporter. In addition, the functional activities of cells whose differentiation and maturation are dependent on the retinoids and the RXR/RAR-PPARγ (RXR/RAR-peroxisome proliferator activated receptor-gamma) signaling pathways (for example, macrophages and dendritic and FOXP3^+ ^T cells) will be altered due to altered development, ultimately affecting antigen presentation and balanced production of regulatory cytokines.

## Conclusion

Identifying gene products that are differentially expressed in the *Aec2 *SjS susceptibility subregion defined by the new RI lines is moving us closer to identifying specific candidate genes involved in the onset and development of SjS-like disease. Based on our current data, our focus is turning to *Ox40L *as an effective candidate gene for the development of SjS. The future application of genetic knockout mice and/or short interfering RNA will permit us to further our understanding of the potential role of *Ox40L *in SjS and, more importantly, to translate its relevancy to human SjS.

## Abbreviations

Aec: autoimmune exocrinopathy; ANA: anti-nuclear autoantibody; IL: interleukin; INF-γ: interferon-gamma; LF: lymphocytic foci; MHC: major histocompatibility complex; NOD: nonobese diabetic; PBS: phosphate-buffered saline; QTL: quantitative trait loci; RAR: retinoic acid receptor; RI: recombinant inbred; RXR: retinoid × receptor; SjS: Sjögren syndrome; SOAT-1: sterol O-acyltransferase-1; TNFSF4: tumor necrosis factor ligand superfamily member 4; T_reg_: T regulatory.

## Competing interests

The authors declare that they have no competing interests.

## Authors' contributions

ABP performed the genotyping of mice and assisted with the study design and manuscript preparation. JT carried out histological analysis. JGC, LC, and JN performed saliva collections and disease profilings of the mice. BHL helped with manuscript preparation. CQN participated in the design of the study, ANA staining, saliva collections, data analyses, and manuscript preparation. All authors read and approved the final manuscript.
